# Discordance between Liver Biopsy and FibroTest in Assessing Liver Fibrosis in Chronic Hepatitis B

**DOI:** 10.1371/journal.pone.0055759

**Published:** 2013-02-06

**Authors:** Mi Sung Park, Beom Kyung Kim, Jae Yoeun Cheong, Dong Joon Kim, Jun Yong Park, Do Young Kim, Sang Hoon Ahn, Kwang-Hyub Han, Chae Yoon Chon, Seung Up Kim

**Affiliations:** 1 Department of Internal Medicine, Yonsei University College of Medicine, Seoul, Korea; 2 Institute of Gastroenterology, Yonsei University College of Medicine, Seoul, Korea; 3 Liver Cirrhosis Clinical Research Center, Seoul, Korea; 4 Department of Gastroenterology, Ajou University School of Medicine, Suwon, Korea; 5 Department of Internal Medicine, Hallym University College of Medicine, Chuncheon, Korea; 6 Brain Korea 21 Project for Medical Science, Seoul, Korea; The University of Hong Kong, Hong Kong

## Abstract

**Background and Aims:**

The FibroTest (FT) demonstrated excellent diagnostic performance in the prediction of liver fibrosis in patients with chronic hepatitis B (CHB). Here, we aimed to identify predictors of discordance between FT and liver biopsy (LB) in Asian patients with CHB.

**Methods:**

Consecutive patients with CHB who underwent both LB and FT on the same day between 2007 and 2010 were recruited from three medical institutes. Laboratory evaluations including specific parameters for calculating FT score, such as α2-macroglobulin, apolipoprotein A1, haptoglobin, γ-glutamyl transpeptidase, and total bilirubin levels, were obtained. The Batts and Ludwig scoring system was used for histological analysis.

**Results:**

A total of 330 patients (200 male and 130 female) were analyzed. Discordances of at least two fibrosis stages between FT and LB were observed in 30 (9.1%) patients; using FT, fibrosis was underestimated in 25 patients and overestimated in 5 patients with reference to LB. Patients with discordance had a higher proportion of F3–4 (*P*<0.001) and F4 (*P* = 0.012) compared with those with nondiscordance. The discordance rate was significantly higher in those with F3–4 than those with F1–2 (15.4% vs. 3.0%, *P*<0.001). Multivariate analysis demonstrated F3–4 at LB as the only independent factor for discordance (*P*<0.001; odds ratio 5.95). After adjusting fibrosis stages, neither necroinflammatory activity on histology nor serum ALT level influenced FT values independently.

**Conclusion:**

Advanced fibrosis stage (F3–4) is the sole factor of discordance between FT and LB in Asian patients with CHB.

## Introduction

Accurate assessment of the severity of liver fibrosis in patients with chronic hepatitis B (CHB) is necessary not only to predict the long-term clinical course, but also to determine whether and when to begin antiviral therapy. To-date, liver biopsy (LB) has been the gold standard for assessing liver fibrosis. However, it is often limited because of its invasiveness, cost, risk of complications, poor acceptance, lack of availability of expert practitioners, and intra/inter-observer variability [1], [2]. These factors make sequential LBs unfeasible, especially when repeated examinations are required to monitor the response to antiviral or anti-fibrotic treatment. Consequently, these limitations have stimulated research into noninvasive approaches. So far, two kinds of noninvasive surrogates have been identified: (1) ultrasound-based methods such as transient elastography [3], [4] or acoustic radiation force impulse (ARFI) [5], and (2) biochemical marker-based methods such as the aspartate aminotransferase (AST)-to-platelet ratio index (APRI) [6], AST-to-alanine aminotransferase (ALT) ratio [7], and FibroTest (FT) (BioPredictive, Paris, France) [8], all of which combine several biochemical parameters [9].

Of these, the FT was first proposed by Poynard *et al*. [8] in 2001. It is a scoring algorithm that uses a panel of five biochemical markers, including α2-macroglobulin, apolipoprotein A1, haptoglobin, γ-glutamyl-transpeptidase (GGT), and total bilirubin to assess liver fibrosis adjusted by age and gender. Since FT was initially investigated in Caucasian populations with chronic hepatitis C (CHC), its performance as a surrogate marker for LB has been studied extensively and has shown a good correlation with histological liver fibrosis stage [10]–[12]. In addition, a recent study reported that FT can also predict liver fibrosis with comparable accuracy to transient elastography in Asian patients with CHB [13].

However, such indirect methods as noninvasive surrogates for LB, including the FT, are always subject to discordance compared with fibrosis staging by LB. Thus, identification of factors associated with the discordance between noninvasive surrogates and LB may enable us to determine the optimal method among several noninvasive tests. Furthermore, if the effects of other clinical variables (*i*.*e*., necroinflammatory activity) on FT values other than the fibrosis stage itself are elucidated, the optimal timing for evaluation of the intrahepatic fibrotic burden could be better determined, ultimately avoiding underestimation or overestimation of the real fibrotic burden.

However, to our knowledge, investigations of these issues are scarce so far, especially for the FT. In this study, we aimed to identify predictors of discordance between LB and the FT and to investigate the potential effects of necroinflammation on FT values in Asian patients with CHB.

## Patients and Methods

### Patients

Consecutive patients with CHB who underwent both LB and the FT on the same day at Severance Hospital, Yonsei University College of Medicine, Seoul, Korea, Ajou University School of Medicine, Suwon, Korea, and Hallym University College of Medicine, Chuncheon, Korea between January 2007 and December 2010 were considered eligible for this study. LB was performed to assess the severity of fibrosis and inflammation prior to treatment.

The exclusion criteria were as follows: previous history of antiviral therapy; history of hepatocellular carcinoma treatment at the time of LB; LB specimen shorter than 20 mm; coinfection with human immunodeficiency virus or hepatitis C or D virus; alcohol ingestion in excess of 40 g/day for more than 3 years; and Child-Pugh classification of B or C.

The study was performed in accordance with the ethical guidelines of the 1975 Declaration of Helsinki. Written informed consent was obtained from each participant or responsible family member after possible complications of the diagnostic procedures had been fully explained.

### Serum Biochemical Markers and FT Score Calculation

All laboratory data including specific parameters for calculating the FT score, such as α2-macroglobulin level, apolipoprotein A1 level, haptoglobin level, γ-GGT level, and total bilirubin level, were obtained on the same day as the FT and LB. BioPredictive kindly provided the complimentary service for calculation of FT score. The discriminant cutoff values for fibrosis stage as estimated by the FT were based on a previous study (F1, FT <0.32; F2, 0.32≤ FT <0.52; F3, 0.52≤ FT <0.68; F4, FT ≥0.68) [13].

### Liver Biopsy Examination

Percutaneous LB was performed using a 16G disposable needle. The LB specimens were fixed in formalin and embedded in paraffin. Sections 4 mm thick were then stained with hematoxylin and eosin and Masson’s trichrome. All liver tissue samples were evaluated by an experienced hepatopathologist in each institute blinded to the patients’ clinical histories. Specimens that were shorter than 17 mm and considered by the pathologists to be unsuitable for fibrosis assessment were excluded from the analysis. Liver histology was evaluated semiquantitatively according to the Batts and Ludwig scoring system [14]. Fibrosis was staged on a 0–4 scale: F0, no fibrosis; F1, portal fibrosis; F2, periportal fibrosis; F3, septal fibrosis; and F4, cirrhosis. Significant fibrosis was defined as F2 or more, advanced fibrosis as F3 or more, and cirrhosis as F4.

### Statistical Analysis

Continuous variables of patients with discordance and those without were compared with independent *t*-tests or Mann-Whitney U tests, as appropriate. The chi-squared or Fisher’s exact tests were used for categorical variables. Based on previous studies [15]–[17], discordance was defined as a discordance of at least two stages between fibrosis stages determined by the FT and LB. Subsequent multivariate analysis using binary logistic regression analyses was performed to identify independent factors related to discordance between fibrosis stage of the FT and LB. Odds ratios (ORs) and corresponding 95% confidence intervals (CIs) were also indicated.

Statistical analyses were performed using the SPSS software (ver. 18.0; SPSS Inc., Chicago, IL, USA). In all analyses, a value of *P*<0.05 was taken to indicate statistical significance.

## Results

### Baseline Characteristics of the Study Population

**Figure 1:**
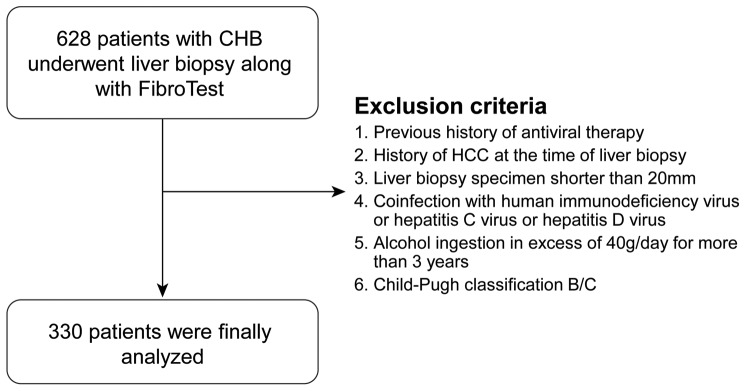
Flow chart describing the selection of the study population. CHB; Chronic hepatitis B, HCC; hepatocellular carcinoma.

A total of 628 consecutive patients were screened for possible inclusion in the study. Based on the above exclusion criteria, a total of 330 patients (200 male and 130 female) were finally analyzed (Figure 1).

Baseline characteristics of study cohort are summarized in Table 1. The median age was 44 years, and male gender predominated (n = 200, 60.6%). The median AST and ALT levels were 42 IU/L (range, 12–385) and 51 IU/L (range, 5–551), respectively. All patients had well-preserved liver functions with Child-Pugh class A.

**Table 1:** Baseline characteristics (n = 330).

Variables	Value
**Demographic data**	
Age (years)	44 (20–83)
Male gender	200 (60.6)
Body mass index (kg/m^2^)	23.4 (15.1–35.8)
Obesity (≥30 kg/m^2^)	9 (2.7)
**Laboratory data**	
Serum albumin (g/dL)	4.3 (2.9–5.4)
Total bilirubin (mg/dL)	0.7 (0.2–2.0)
Aspartate aminotransferase (IU/L)	42 (12–385)
Alanine aminotransferase (IU/L)	51 (5–551)
Prothrombin time (INR)	1.1 (0.91–1.25)
Platelet count (10^9^/L)	186 (80–489)

Values are expressed as median (range) or no. (%).

INR, international normalized ratio.

### Liver Histology and Corresponding FT Values

**Figure 2:**
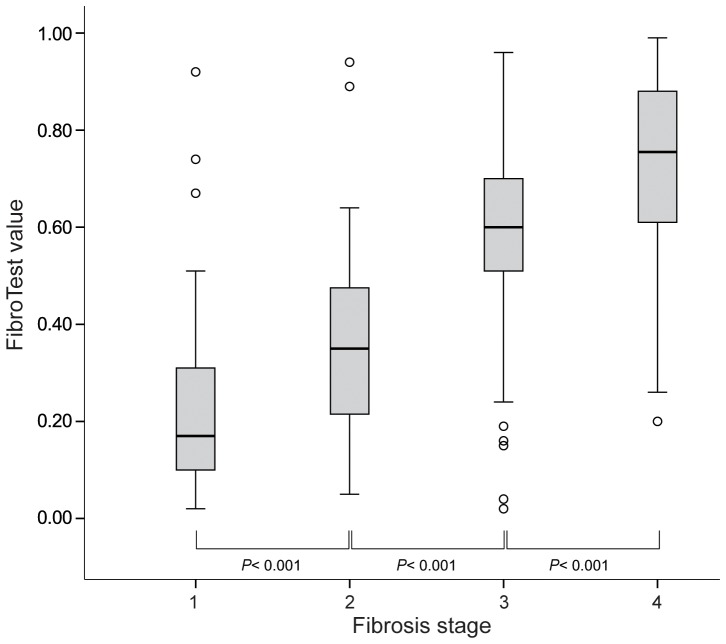
Box plots of FT values according to fibrosis stage. Mean FT values increase significantly as fibrosis stage increase (0.17 for F1 (range, 0.02–0.92), 0.35 for F2 (range, 0.05–0.94), 0.6 for F3 (range, 0.02–0.96), and 0.76 for F4 (range, 0.2–0.99), all *P*<0.001 among fibrosis stages).

The median length of LB samples was 22 mm (range, 20–25). The distribution of FT values according to maximal inflammatory activity in each fibrosis stage is shown in Table 2. The mean FT values increased significantly as fibrosis stage increased (0.23±0.17 for F1, 0.35±0.18 for F2, 0.59±0.21 for F3, and 0.71±0.21 for F4; all *P*<0.001 between each fibrosis stage) (Table 2 and Figure 2).

**Table 2:** Liver histology and corresponding FT values (n = 330).

Fibrosis		FT value*	Maximal activity^a^		FT value*
Stage	no. (%)	Mean ± SD	Grade	no.	Mean ± SD
1	65 (19.7)	0.23±0.17	1	32	0.22±0.18
			2	26	0.24±0.18
			3	6	0.21±0.11
			4	1	0.07
2	103 (31.2)	0.35±0.18	1	11	0.35±0.15
			2	51	0.32±0.18
			3	27	0.38±0.19
			4	14	0.40±0.14
3	84 (25.5)	0.59±0.21	1	6	0.49±0.16
			2	28	0.58±0.21
			3	39	0.61±0.20
			4	11	0.58±0.24
4	78 (23.6)	0.71±0.21	1	7	0.75±0.24
			2	53	0.72±0.21
			3	16	0.68±0.21
			4	2	0.76±0.11

FT values* are described as mean ± standard deviation.

Maximal activity^a^ grade was defined as the higher one between lobular and periportal activity.

Using discriminant cutoff values to predict each fibrosis state (F1, F2, F3, and F4) by the FT (values were suggested in a previous study [13]), a discordance of at least two stages (gray) between the FT and LB was observed in 30 (9.1%) patients (Table 3). On the whole, using the FT, fibrosis stage was underestimated in 25 patients and overestimated in 5 patients with reference to LB (Table 3).

**Table 3:** Distribution of fibrosis stages according to histology and FT.

Fibrosis stage estimated by histology	Fibrosis stage estimated by FT			
	F1	F2	F3	F4
	FT<0.32	0.32≤FT<0.52	0.52≤FT<0.68	FT≥0.68
F1 (n = 65)	50	12	**1**	**2**
F2 (n = 103)	46	40	45	**2**
F3 (n = 84)	**11**	12	36	25
F4 (n = 78)	**8**	**6**	10	54
Total (n = 330)	115	70	92	83

### Factors Affecting the Discordance between Histology and FT Values

**Figure 3:**
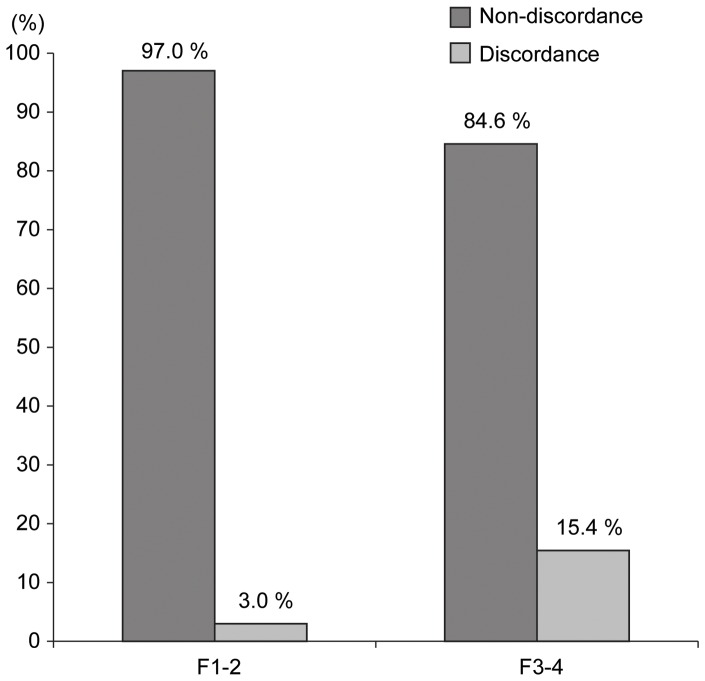
Percentage of patients with non-discordance and those with discordance in fibrosis stage 1–2 (F1–2) vs. **fibrosis stage 3–4 (F3–4).** Patients with F3–4 had the significantly higher proportion of discordance than those with F1–2 (15.4% vs. 3.0%, *P*<0.001).

When we compared baseline characteristics of patients with nondiscordance and those with discordance, patients with discordance had a higher proportion of F3–4 (*P*<0.001) and F4 (*P* = 0.012) than those with nondiscordance, with statistical significance (Table 4). The discordance rate was significantly higher in patients with F3–4 than in those with F1–2 (15.4% vs. 3.0%, respectively; *P*<0.001) (Figure 3).

**Table 4:** Comparison of patients with non-discordance and those with discordance.

Variables	Patients with non-discordance	Patients with discordance	*P* value
	(n = 300, 90.9%)	(n = 30, 9.1%)	
**Clinical variables**			
Age (years)	44 (20–83)	43 (24–65)	NS
Male gender	186 (62.0)	14 (46.7)	NS
Body mass index (kg/m^2^)	23.5 (15.1–35.8)	23.1 (18.7–31.3)	NS
Obesity (≥30 kg/m^2^)	7 (2.3)	2 (6.7)	NS
Aspartate aminotransferase (IU/L)	42 (16–385)	38 (12–70)	NS
Alanine aminotransferase (IU/L)	49 (7–551)	55 (5–157)	NS
Platelet count (10^9^/L)	186 (80–489)	175 (100–283)	NS
**Fibrosis stage**			
F2–4	238 (79.3)	27 (90.0)	NS
F3–4	137 (45.7)	25 (83.3)	<0.001
F4	63 (21.0)	14 (46.7)	0.012
**Maximal activity** ^a^			
A2–4	249 (83.0)	26 (86.7)	NS
A3–4	107 (35.7)	9 (30.0)	NS
A4	27 (9.0)	4 (13.3)	NS

Maximal activity^a^ grade was defined as the higher one between lobular and periportal activity.

Values are expressed as median (range) or no. (%).

Subsequent multivariate analysis demonstrated that F3–4 at the time of LB was selected as the only independent factor for discordance between the two tests showing a positive correlation (*P*<0.001; adjusted OR, 5.95; 95% CI 2.22–15.96) (Table 5).

**Table 5:** Factors affecting discordance between FT and histology.

	Univariate	Multivariate	
	*P* value	*P* value	Adjusted odds ratio (95% confidence interval)
**Clinical data**			
Age (years)	NS		
Male gender	NS		
Body mass index (kg/m^2^)	NS		
Aspartate aminotransferase (IU/L)	NS		
Alanine aminotransferase (IU/L)	NS		
Platelet count (10^9^/L)	NS		
**Fibrosis stage**			
F4 (vs. F1–3)	0.003		
F3–4 (vs. F1–2)	<0.001	<0.001	5.95 (2.22–15.96)
F2–4 (vs. F1)	NS		
**Maximal activity** ^a^			
A4 (vs. A1–3)	NS		
A3–4 (vs. A1–2)	NS		
A2–4 (vs. A1)	NS		

Maximal activity^a^ grade was defined as the higher one between lobular and periportal activity.

NS, not significant.

### Influence of Histological Necroinflammation and ALT Level on FT Values

Although significant differences in necroinflammatory activity and serum ALT level between patients with discordance and those with nondiscordance were not detected (Table 4), previous studies of ultrasound-based tests such as transient elastography and ARFI [5], [16], [18] reported overestimation of fibrosis due to secondary effects of high ALT levels and/or necroinflammation. Thus, we further analyzed the influence of histological necroinflammation and ALT level in our cohort.

**Figure 4:**
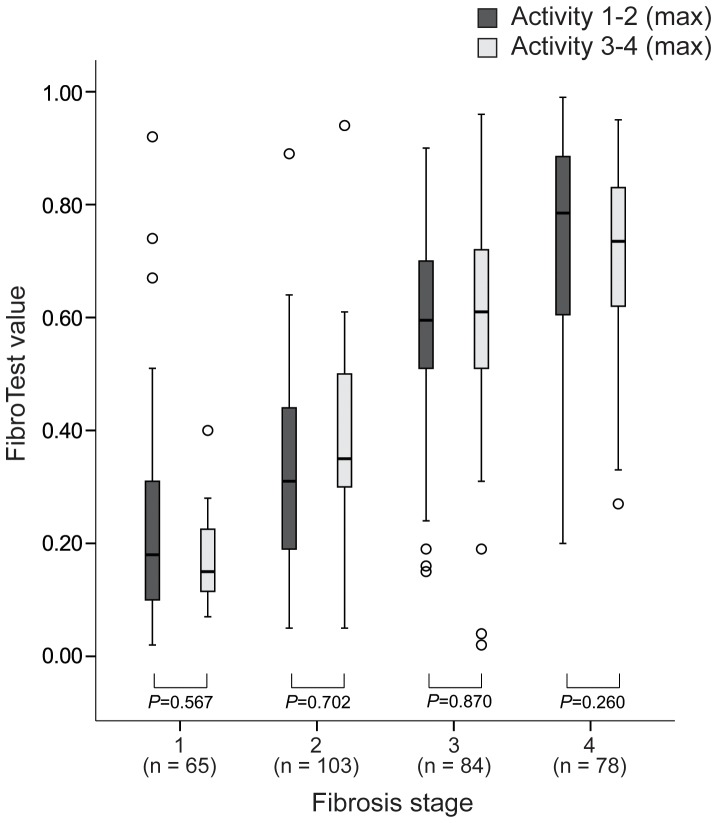
Distribution of FT values according to fibrosis stage and maximal activity grade 1–2 vs. 3–4. FT values were statistically equivalent between maximal activity grade 1–2 and 3–4 when stratified by each fibrosis stage (all *P*>0.05).

**Figure 5:**
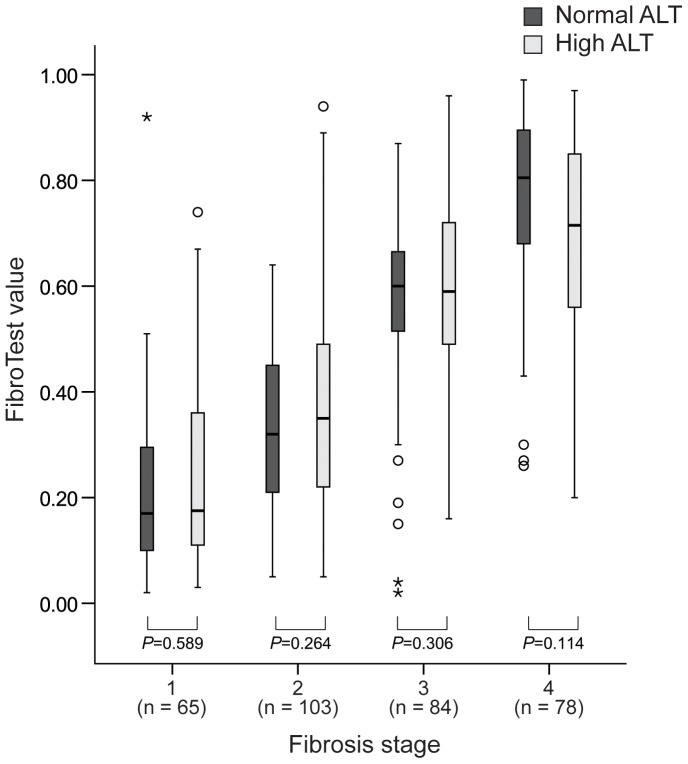
FT values between patients with normal ALT and those with high ALT in each fibrosis stage. FT values were statistically equivalent between two groups (all *P*>0.05).

FT values between patients with maximal activity of grades 1–2 and 3–4 were statistically equivalent in each fibrosis stage (all *P*>0.05, Figure 4). In addition, there was no significant difference in FibroTest value between patients with normal ALT and those with high ALT in each fibrosis stage (all *P*>0.05, Figure 5).

### Factors Affecting Discordance Based on External Cutoff Values

When we used the external cutoff values from BioPredictive (0.49, 0.59, and 0.75 for F ≥2, F ≥3, and F >4, respectively), discordances between the FT and LB were found in 41 (12.4%) patients; 36 patients were underestimated using the FT, whereas 5 patients were overestimated. Similarly, a higher proportion of patients with an advanced fibrosis stage (F3–4) were identified in patients with discordance (*P*<0.001). On multivariate analysis, advanced fibrosis stage (F3–4) was the only predictor positively affecting discordance between the FT and LB (*P*<0.001; adjusted OR 9.31, 95% CI 3.55–24.42) (Table S1).

## Discussion

The FT is known to show good diagnostic performance with reference to fibrosis stages of LB in not only the Caucasian population with CHC, but also Asian patients with CHB [10]–[13]. However, noninvasive surrogates for LB including the FT, no matter how they achieve their excellent diagnostic performance, are inherently subject to discordance compared with the fibrosis staging by LB primarily because noninvasive surrogates indirectly assess the total fibrotic burden instead of the architectural changes in liver parenchyma, which is the major factor in histological evaluation [19]. Nevertheless, because LB is the current gold standard, identification of factors associated with the discordance between noninvasive surrogates and LB is an important prerequisite for accurate assessment of the intrahepatic fibrotic burden. However, investigations on this issue are scarce so far, especially for the FT. In addition, data on the influence of necroinflammation on the FT value are lacking, which can be an important issue for two reasons: first, patients in whom LB is required usually have evidence of necroinflammation along with fibrotic burden; and second, probable effects (*i*.*e*., overestimation of fibrosis stage) of necroinflammation have been reported for several surrogate tests [5]. Therefore, our findings will assist formulation of guidelines for optimal evaluation of the intrahepatic fibrotic burden, ultimately avoiding underestimation or overestimation of the real fibrotic burden.

To our knowledge, this is the first study to investigate the prevalence of the discordance between LB and the FT and its predictors in Asian patients with CHB in a relative large cohort from three medical institutes. Advanced fibrosis (F3–4) at LB is a sole factor attributed to discordance between fibrosis stages by LB and the FT, indicating a greater chance of agreement between LB and the FT when patients with CHB show F0–2 at the time of LB. Indeed, most discordance in this study was caused by underestimation of liver fibrosis by the FT in patients with F3–4 (11 cases of F3 and 14 of F4 of 30 discordant cases). Furthermore, we analyzed the additional influence of histological necroinflammatory activity and serum ALT level on FT values in each fibrosis stage; neither affected either the likelihood of discordance or FT value itself. Interestingly, when we used the external cutoff values from BioPredictive, an advanced fibrosis stage of F3–4 was selected again as the only predictor of discordance.

In contrast to the FT, for transient elastography, a fibrosis stage of F0–2 is the only factor that predicts significant discordance with reference to LB in Asian patients with CHB, and necroinflammation (*i*.*e*., serum ALT level more than twice the upper normal limit) has been identified as a significant confounder, which nonspecifically increases liver stiffness values and thus leads to overestimation of the fibrosis stage [15]–[18], [20]. Therefore, with active necroinflammation or ALT flares, measuring liver stiffness using transient elastography should be delayed after stabilization of necroinflammation and normalization of serum ALT to exclude such undesirable overestimating effects [21]. Such contradictory results of the FT and transient elastography are noteworthy in that they might be mutually complementary in assessing liver fibrosis in terms of combining the FT with transient elastography to enhance the diagnostic accuracy.

However, paradoxically, a histological stage of F0–2 favoring the use of the FT or F3–4 favoring the use of transient elastography cannot be revealed without LB. Therefore, the optimal diagnostic algorithm for use of these two noninvasive surrogates remains unresolved. The French guideline for diagnosis of uncomplicated cirrhosis [22] is an example in which the FT and transient elastography are recommended as the first-line noninvasive diagnostic tools for liver fibrosis. In these guidelines, the fibrosis stage can be confirmed without LB for a given patient if the respective fibrosis stage based on the FT and transient elastography coincides, whereas LB is recommended when there is a discordant result in fibrosis staging between the FT and transient elastography. However, although this diagnostic strategy is based mostly on accumulated data from studies on CHC, it seems reasonable to combine the FT with transient elastography for enhanced diagnostic accuracy in assessing liver fibrosis in patients with CHB when we consider the relatively lower performance of transient elastography in patients with CHB than in those with CHC [4]. The paucity of data regarding FT in patients with CHB and the increase in cost of addition of the FT should also be addressed in future studies.

This study has several strengths. First, a relatively large number of subjects from three medical institutes were consecutively enrolled, and the study population was homogenous and representative of patients with CHB generally seen in clinical practice. Second, because we recruited only patients who underwent baseline blood tests on the same day as LB, potential bias due to the gap between the two tests might have been prevented. Third, by demonstrating predictors of discordance between the FT and LB and proposing a rationale for combining several noninvasive surrogates to enhance the diagnostic accuracy, our findings will facilitate future studies that aim to propose a new noninvasive diagnostic algorithm for liver fibrosis in Asian patients with CHB.

However, we are also aware of several limitations of our study. First, there were no subjects with F0 fibrosis. Because three medical centers in this study are tertiary referral hospitals and one of the largest medical centers in Korea, patients with relatively more advanced disease are likely to be referred for close follow-up. This might have resulted in a selection bias and eventually a spectrum bias because the diagnostic performance of a given noninvasive test tends to increase in general in a cohort with high disease prevalence. Thus the diagnostic performances of FT might have been overestimated in our cohort. Therefore, another independent external validation study in a population with minimal fibrotic burden should be performed to provide more generalizable results in patients with CHB-related chronic liver disease. Second, we could not investigate the clinical role of the FT as a noninvasive monitoring tool to predict dynamic changes in liver fibrosis because a follow-up FT was not available. Third, although we identified predictors of discordance, it is unclear whether the long-term prognosis of patients with discordance differs from that of patients with nondiscordance. In addition, whether we should follow the results of the FT or LB to start treatment intervention and predict the prognosis of a given patient is unclear. Clarification of these issues requires longitudinal studies using solid end-points such as hepatocellular carcinoma or liver-related death.

In conclusion, advanced fibrosis stage (F3–4) showed a positive correlation with discordance between LB and the FT in patients with CHB. Furthermore, necroinflammation and ALT levels were not factors predictive of accuracy. Future studies should focus on methods of enhancing the diagnostic accuracy of the FT in clinical practice.

This manuscript was checked by a professional editing service (www.textcheck.com, certificate link: http://www.textcheck.com/certificate/jNAqDE).

## Supporting Information

Table S1 **Factors affecting discordance between FT and histology by cutoff values from BioPredictive.**
(DOCX)
