# Supplementary material for: Discordance between Liver Biopsy and FibroTest in Assessing Liver Fibrosis in Chronic Hepatitis B
Source: PLoS One. 2013 Feb 6;8(2):e55759. doi: 10.1371/journal.pone.0055759 (PMC3566034; doi:10.1371/journal.pone.0055759)
Supplement: Table S1 — Factors affecting discordance between FT and histology by cutoff values from BioPredictive. (DOCX) [file pone.0055759.s001.docx]

| **Supplementary table 1.** Factors affecting discordance between FT and histology by cutoff values from BioPredictive | | | | |
| --- | --- | --- | --- | --- |
|  | Univariate |  | Multivariate | |
|  | *P* value |  | *P* value | Adjusted odds ratio  (95% confidence interval) |
| Age (years) | NS |  |  |  |
| Male gender | NS |  |  |  |
| Body mass index (kg/m^2^) | NS |  |  |  |
| Aspartate aminotransferase (IU/L) | NS |  |  |  |
| Alanine aminotransferase (IU/L) | NS |  |  |  |
| Platelet count (10^9^/L) | NS |  |  |  |
| F3-4 (vs. F1-2) | <0.001 |  | <0.001 | 9.31 (3.55 - 24.42) |
| A3-4 (vs. A1-2)^a^ | NS |  |  |  |
| Maximal activity^a^ grade was defined as the higher one between lobular and periportal activity. | | | | |
| NS, not significant. | | | | |
